# First Molecular Characterization of *Siphoviridae*-Like Bacteriophages Infecting *Staphylococcus hyicus* in a Case of Exudative Epidermitis

**DOI:** 10.3389/fmicb.2021.653501

**Published:** 2021-06-30

**Authors:** Julia Tetens, Sabrina Sprotte, Georg Thimm, Natalia Wagner, Erik Brinks, Horst Neve, Christina Susanne Hölzel, Charles M. A. P. Franz

**Affiliations:** ^1^Institute of Animal Breeding and Husbandry, Kiel University, Kiel, Germany; ^2^Department of Microbiology and Biotechnology, Max Rubner-Institut, Federal Research Institute of Nutrition and Food, Kiel, Germany

**Keywords:** bacteriophages, *Staphylococcus hyicus*, *Siphoviridae*-like, exudative epidermitis, pig

## Abstract

Exudative epidermitis (EE), also known as greasy pig disease, is one of the most frequent skin diseases affecting piglets. Zoonotic infections in human occur. EE is primarily caused by virulent strains of *Staphylococcus* (*S*.) *hyicus*. Generally, antibiotic treatment of this pathogen is prone to decreasing success, due to the incremental development of multiple resistances of bacteria against antibiotics. Once approved, bacteriophages might offer interesting alternatives for environmental sanitation or individualized treatment, subject to the absence of virulence and antimicrobial resistance genes. However, genetic characterization of bacteriophages for *S. hyicus* has, so far, been missing. Therefore, we investigated a piglet raising farm with a stock problem due to EE. We isolated eleven phages from the environment and wash water of piglets diagnosed with the causative agent of EE, i.e., *S. hyicus*. The phages were morphologically characterized by electron microscopy, where they appeared *Siphoviridae*-like. The genomes of two phages were sequenced on a MiSeq instrument (Illumina), resulting in the identification of a new virulent phage, PITT-1 (PMBT8), and a temperate phage, PITT-5 (PMBT9). Sequencing of three host bacteria (*S. hyicus*) from one single farm revealed the presence of two different strains with genes coding for two different exfoliative toxin genes, i.e., *exh*A (2 strains) and *exh*C (1 strain). The *exh*C-positive *S. hyicus* strain was only weakly lysed by most lytic phages. The occurrence of different virulent *S. hyicus* strains in the same outbreak limits the prospects for successful phage treatment and argues for the simultaneous use of multiple and different phages attacking the same host.

## Introduction

Greasy pig disease, also known as exudative epidermitis, is a common skin disease in young piglets and weaners causing significant morbidity and mortality in naïve herds. The main cause of this disease are pathogenic strains of *Staphylococcus* (*S*.) *hyicus* that produce exfoliative toxins (Foster, 2012). *S. hyicus* is part of the commensal skin microbiome of healthy pigs (Nagase et al., 2002). Virulence is closely associated with the production of exfoliative toxins that target the cells of the *Stratum spinosum* in the epidermis (Nishifuji et al., 2008). Originally, six exfoliative toxins (ExhA, ExhB, ExhC, ExhD, SHETA, and SHETB) were described (Sato et al., 2000; Ahrens and Andresen, 2004) and recently a new exfoliative toxin (ExhE) has additionally been characterized (Imanishi et al., 2019). Although *S. hyicus* can directly penetrate the skin, skin lesions—resulting for example from biting—are primarily the starting point of greasy pig disease (Frana and Hau, 2019). Diseased piglets show skin lesions at first on the head and on hairless skin of the medial aspect of legs (Andrews, 1979). Lesions can progress to cover the whole body with a greasy brown exudate that may lead to dehydration and animal death (Foster, 2012). Antibiotics are regularly used for treatment, but the presence of *S. hyicus* isolates that exhibit broad-spectrum resistance to antimicrobials have been increasingly reported (Aarestrup and Jensen, 2002; Futagawa-Saito et al., 2009; Park et al., 2013b). This is alarming not only from a veterinary, but also from a One Health perspective and requires alternative strategies for therapy as well as prevention. To protect piglets from this disease, vaccination of gestating sows using attenuated strains isolated from the affected herd is a common practice. Nevertheless, this autogenous vaccination is apparently not able to protect all piglets against greasy pig disease. Furthermore, scientific evidence for the efficacy of autogenous vaccines against *S. hyicus*, as described at least once by Arsenakis et al. (2018), is scarce.

Bacteriophages (phages), defined as viruses that infect and kill bacteria, might offer an alternative opportunity for prevention and therapy of diseases (Gigante and Atterbury, 2019). Virulent or lytic phages bind to specific receptors on the bacterial cell surface, inject their genetic material and then hijack the bacterial replication machinery to produce the next generation of phage progeny and subsequently lyse the host cell (Howard-Varona et al., 2017). Temperate phages, on the other hand, do not primarily produce phage progeny and lysis of host cells, as they integrate their genetic material into the bacterial genome and reproduce their genome passively (vertically) from mother to daughter bacterial cells (Salmond and Fineran, 2015). Compared with antibiotics, the risk to develop phage resistance is much lower, as phages may overcome mutations in bacteria that lead to resistance mechanisms (Labrie et al., 2010). In general, phages reveal high specificity. Thus, phages only kill target bacterial species or even strains (Rakhuba et al., 2010) but do not destroy the host's commensal microbiota. In reverse conclusion, this implies a need to extensively screen for an effective phage for every pathogen considered (Rohde et al., 2018).

In this study, we investigated a piglet raising farm with greasy pig disease as a stock problem in order to isolate, identify and characterize bacteriophages for *S. hyicus*. To our knowledge, this is the first attempt to characterize bacteriophages for *S. hyicus* using molecular biological methods.

## Methods, Techniques

### Sample Collection

#### Isolation and Selection of *Staphylococcus hyicus*

Samples were scraped from the skins of 25 weaners (42–63 days of age and 19–26 kg of body weight) which showed typical clinical signs of greasy pig disease using sterile cotton swabs. All swabs were directly streaked out on Columbia blood agar (7% sheep blood; Oxoid, Wesel, Germany) and incubated at 37°C for 24 h.

#### Phage Isolation

Five different environmental samples were collected on the farm in parallel with sampling for *S. hyicus*. Two of these samples were liquid pig manure originating from two different pig houses on the corresponding farm. Another sample originated from washing water of a heavily infected piglet and a further sample originated from wastewater of a slatted floor. The last sample was a combination of washing water of an infected piglet and wastewater of a slatted floor. Two hundred and fifty milliliter of all five environmental samples were first centrifuged (14,300 × *g*, 15 min, 4°C) and then filtered through a 0.45 μm membrane filter (Filtropur S, Sarstedt, Nürnbrecht, Germany). A 5 ml aliquot filtrate of each of the five environmental samples was mixed with 100 μl CaCl_2_ (1 M) and 100 μl of a fresh overnight culture of the host bacterium *S. hyicus* (described in Isolation and Selection of *Staphylococcus hyicus* & Bacteriological Investigations). The mixture was incubated overnight at 37°C, then centrifuged at 18,000 × *g* for 15 min to remove residual bacterial cells. The supernatant was filtered again using a 0.45 μm membrane filter.

### Bacteriological Investigations

#### Identification of *S. hyicus*

Colonies were picked from Columbia blood agar and incubated on Baird-Parker and Chocolate agar (Oxoid) at 37°C for 24 h. Isolates suspected as *S. hyicus* on the basis of hemolytic reaction on Chocolate agar and typical growth on Baird-Parker-agar (i.e., black, shiny, convex colonies, lack of clear zones, no precipitation), were chosen for Gram-staining and basic biochemical standard tests (i.e., catalase-test, oxidase-test, clumping-factor test). Five strains presenting Gram-positive, catalase-positive, oxidase-negative, clumping-factor- and coagulase-negative cocci were chosen for species confirmation PCR.

#### Antibiotic Susceptibility Tests

All five *S. hyicus* strains were tested for cefoxitin-resistance using disk diffusion in order to indicate a methicillin-resistant phenotype. Disk diffusion tests were performed according to EUCAST instructions (http://www.eucast.org). Strains were suspended in 0.9% NaCl solution at a density equivalent to a 0.5 McFarland standard and spread in dense lines in two rectangular and one diagonal layer on Mueller-Hinton agar plates using sterile cotton swabs (Oxoid, Wesel, Germany). Antibiotic disks containing cefoxitin (30 μg) were loaded onto the plates. After incubation for 18 h, the inhibition zone diameters were measured upon inspection against a dark background illuminated with reflected light. Zone diameters were interpreted based on EUCAST breakpoints for *Staphylococcus* spp.

### Phage Isolation and Characterization

#### Spot Testing

To determine the presence of phages, spot tests were performed with all five filtrates and five PCR-confirmed *S. hyicus* strains using the double-layer agar method. In detail, 300 μl of a fresh overnight culture of each *S. hyicus* strain were mixed with 100 μl of 40 mM CaCl_2_ and 3 ml of Caso-soft agar [Caso broth with 0.7% (w/w) agar] held at 50°C and immediately poured onto plate count agar. After the solidification of the soft agar, 100 μl of each filtrate, respectively, was spotted in the middle of the plate on the bacterial lawn and all 25 plates were incubated at 37°C for 24 h. After incubation, plates were observed for the presence of lysis zones. When lysis zones appeared, they were carefully scraped off and resuspended in 4 mL of SM-buffer [0.58% NaCl, 0.25% MgSO_4_ × 7H_2_O, 0.24% Tris-HCl (pH 7.4)] for 24 h. Afterwards, the suspension was vortexed and again sterile filtered and stored at 4°C.

#### Plaque Testing

For isolation and purification of bacteriophages, a plaque assay (i.e., double-layer agar method) was performed. For this, 10-fold serial dilutions of the phage filtrates were produced. Subsequently, 100 μl of each of the phage dilutions 10^−2^, 10^−4^, 10^−6^, and 10^−8^ were mixed with 300 μl of a fresh overnight culture of the corresponding host bacterium, 100 μl 40 mM CaCl_2_ and 3 ml of Caso-soft agar held at 50°C and immediately poured onto a plate-count agar plate. After solidification, the soft agar plates were incubated at 37°C for 24 h. Plaques showing different morphologies were picked and pure phage isolates were obtained through three successive single plaque isolation steps.

#### Transmission Electron Microscopy

Phage lysates were first dialyzed for 20 min against modified SM-buffer [20 mM Tris-HCl (pH 7.2), 10 mM NaCl, 20 mM MgSO_4_]. Dialysed phages were adsorbed for 20 min to ultrathin carbon films. After a 20-min fixation with 1% glutaraldehyde and subsequent negative staining with 2% (wt/vol) uranyl acetate, transmission electron microscopy was performed at an accelerating voltage of 80 kV (Tecnai 10; FEI Thermo Fisher Scientific, Eindhoven, The Netherlands). Micrographs were captured with a MegaView G2 CCD camera (Emsis, Muenster, Germany).

#### Host Range

The host range and EOP values of phages PITT 1–PITT 11 were determined for various *S. hyicus* and *S. aureus* isolates from this study, using the plaque assay as described above. In addition, *S. hyicus, S. epidermidis*, and *S. aureus* strains from our own bacterial culture collections and the German Collection of Microorganisms and Cell Cultures (DSMZ, Braunschweig, Germany) were included (DSM 17421, DSM 20459, L2-82, L2-92, L2-93, and L2-152 = DSM 1798; L2-246, L2-248 = DSM 105272, respectively). Strains from our own bacterial culture collections were identified either by MALDI-TOF-MS or 16S-amplicon-sequencing (*S. epidermidis*, primer pair *27F*: AGRGTTYGATYMTGGCTCAG and *1492R*: RGYTACCTTGTTACGACTT). The plaque assay was used to determine the number of plaques produced on the lawns of susceptible *Staphylococcus* strains. The EOP value represents the ratio of plaques obtained with the susceptible test strain when compared to plaques obtained with the isolation strain. All experiments were done in duplicate.

## Molecular Biological Methods

### DNA-Extraction Methods

#### DNA Extraction for Species-Specific PCR

For species-specific PCR, bacterial DNA was extracted by means of a simple lysostaphin-proteinase K-boiling-protocol: from a pure culture of *S. hyicus*, three colonies were picked, suspended in 0.5 ml TE-buffer plus 0.25 ml of lysostaphin and incubated for 1 h at 37°C. After adding 10 μl proteinase K, the suspension was incubated again for 2 h at 56°C. Finally, the suspension was boiled (100°C) for 10 min and was put immediately on ice for 2 min. The suspension was centrifuged at 13,000 × *g* for 3 min and 1 μl of the supernatant was used in the PCR reaction.

#### DNA Extraction for Genome Sequencing of Bacteria

Bacterial DNA for genome sequencing was extracted from early log-phase cultures after 3 h of incubation at 37°C as follows: suspensions were centrifuged 1 min at 16,000 × *g*, the pellet was resuspended in 200 μl Caso broth, transferred to a ZR BashingBead^TM^ Lysis Tube (Zymo Research, Freiburg, Germany) and homogenized in a Precellys 24 device (Bertin Technologies). DNA was then extracted using the Quick-DNA^TM^ Fungal/Bacterial Miniprep Kit (Zymo Research) according to the manufacturer‘s protocol.

#### DNA Extraction From Bacteriophages

For DNA extraction, the Quick-DNA™ Fungal/Bacterial Miniprep Kit was used with some modifications: Several agar plates with phage-derived, confluent lysis were prepared and treated as follows: Plates were floated with 5 ml of SM-buffer and incubated for 3 h at room temperature on an orbital shaker (100 rpm). Next, the buffer-phage suspension was pipetted into two 2-ml tubes, which were centrifuged for 3 min at 8,000 × *g*. After filtration (0.45 μm), the supernatant was centrifuged again for 2.5 h at 14,000 × *g* at 4°C. Pellets were resuspended in 200 μl of SM buffer. RNase and DNase (20 μg ml^−1^ each), respectively, were added for over-night incubation at 37°C. Inactivation of the enzymes was performed for 5 min at 75°C. One milliliter lysis solution from the DNA isolation kit was added and mixed thoroughly for 10 s. Proteinase K was then added at a final concentration of 80 μg ml^−1^ and the sample was incubated first for 30 min at 55°C, then at 65°C for 15 min while inverting the sample 2–3 times. Following this, the sample was mixed with 640 μl isopropanol and loaded onto a DNA-binding column from the kit. For all subsequent steps, the protocol of the manufacturer was followed. Finally, DNA amounts were quantified using a Qubit 3 fluorometer (Invitrogen, Darmstadt, Germany).

#### Species-Specific PCR (*S. hyicus*)

Presumptive strains were confirmed as *S. hyicus* by PCR as described by Voytenko et al. (2006), using primers for the *sod*A gene and two primers for the spacer region between the genes that code for 16S rRNA and 23S rRNA (one specific for *S. hyicus*, one specific for the genus *Staphylococcus*).

#### RAPD-PCR

RAPD-PCR was performed as described elsewhere (Gutiérrez et al., 2011) using random OPL5-primer (5′-ACGCAGGCAC-3′) and phage DNA concentrations of 10 ng per PCR-reaction. PCR-conditions were 4 cycles at 94/20/72°C (45/120/60 s), followed by 26 cycles at 94/36/72°C [5/30 plus (n _cycle_ minus 1), 30 s] and a final elongation at 75°C for 7 min. PCR products were transferred to a 0.8% agarose-gel and separated at 100 V for 55 min.

#### Genome Sequencing and Analysis

Phage DNA was sequenced to characterize the phages with respect to their lytic or temperate life cycle. Genomes of host bacterial strains were sequenced in order to assess their clonal identity and to determine which exfoliative toxin genes were present. Phage DNA was extracted and DNA-concentration was confirmed to be at least 50 ng μl^−1^ using a Qubit 3 fluorometer (Invitrogen, Darmstadt, Germany). The genome was sheared into 450 bp fragments using a Covaris M220 Focused-Ultrasonicator (Covaris, INC, Massachusetts, USA) and the phage DNA library was prepared using a TruSeq Nano DNA LT Library Prep Kit. The MiSeq Reagent Nano Kit v2 (500 cycles) (Illumina, Munich, Germany) was used for sequencing on a MiSeq high throughput sequencer (Illumina). The same library preparation procedure was used for sequencing the bacterial genomes, however, for sequencing these on a MiSeq high throughput sequencer, the MiSeq Reagent Kit v3 (600 cycles) (Illumina) was used. Both sequencing runs produced 2 × 250 bp paired-end reads, which were *de novo* assembled with PATRIC Version 3.5.41 (Wattam et al., 2017). Open reading frames (ORFs) were automatically predicted by PATRIC and afterwards manually analyzed for their putative functions with BLASTP (Altschul et al., 1990) and SMART (Letunic and Bork, 2018). Phage genomes were screened for tRNA-encoding genes with tRNAscan-SE v. 2.0 (Lowe and Chan, 2016) and for acquired virulence and antibiotic resistance genes using ResFinder 4.0 (Bortolaia et al., 2020). Genomes were visualized using Geneious Version 9.1.8 (Kearse et al., 2012). SNP analysis of the bacterial genomes was conducted using CSI Phylogeny 1.4 (Kaas et al., 2014). *S. hyicus* strain ATCC 11249, the closest relative according to BLAST, was used as reference genome. Furthermore the bacterial genomes were analyzed for the presence of exfoliative toxin genes *exh*A, *exh*B, *exh*C and *exh*D using Geneious and primer sequences reported in Ahrens and Andresen (2004). The toxin gene amplicons were compared to the complete gene sequences deposited with the following GenBank accession numbers: ExhA (AF515453); ExhB (AF515454); ExhC (AF515455); and ExhD (AF515456) (Ahrens and Andresen, 2004).

## Results

### Isolation of Bacteria

Samples from 23 of the 25 weaners yielded presumptive *S. hyicus* isolates with typical reactions and characteristic morphology on chocolate agar. Further investigations were performed with five strains from five pigs kept in two different houses and four different compartments. These isolates (07/2 4A, 07/12 1A, 07/12 2A, 83/7 1B, 83/11 1A) were confirmed as *S. hyicus* by species specific PCR. The disk diffusion assay indicated susceptibility for cefoxitin and thus the absence of a methicillin-resistant phenotype.

### Phage Isolation and Morphology

Liquid pig manure samples failed to result in positive spot tests with phage-derived lysis zones. By contrast, all three samples of rinsing water, which were collected from the slatted floor and/or diseased animals, yielded positive spot test results, visible as phage lysis zones.

From seven plates with clear lysis spots, all lysate dilutions (1:100) up to 10^−6^ yielded clear plaques. Two weakly positive reactions (sample C with 07/12 1A and sample C with 07/12 2A) could not be confirmed by plaque assays. Most plaques revealed similar morphology, but finally 11 different plaques with different morphologies could be selected for further investigation (Table 1).

**Table 1:** Origin of the *S. hyicus* phages and host strains used for isolation.

**Combination**		**Rinsing water from**		
		**House 1**		**House 2**
		**A (slatted floor)**	**B (diseased pig)**	**C (slatted floor + diseased pig)**
Bacterial isolate used for primary enrichment	**07/2-4A**	–#	**PITT-1 (PMBT8)**	–
	07/12-1A	–	–	No plaque^*^
	07/12-2A	–	PITT-2, PITT-3	No plaque^*^
	**83/7-1B**	–	PITT-4	**PITT-5 (PMBT9)**
	**83/11-1A**	PITT-10, PITT-11	PITT-8, PITT-9	PITT-6, PITT-7

*# “–” Means negative in spot test, plaque test not done*.

* *Weak lysis zone when spot-tested, not confirmed when plaque-tested*.

*Strains and phages highlighted in **bold** were selected for DNA sequence analysis*.

Based on EOP-analysis, phages showed different lytic activities (Table 2). Phages PITT-4 and PITT-5 lysed only their primary host, while PITT-7 had the broadest host range, efficiently lysing 6 out of 7 strains. PITT-6, PITT-10, and PITT-11 lysed 5 out of 7 strains. Only PITT-6 and PITT-7 were able to lyse *S. hyicus* DSM 17421, while only PITT-7, PITT-10, PITT-11 were able to lyse *S. hyicus* DSM 20459. However, it should be noted that the EOP values were significantly reduced when the titers of these phages were determined on non-host strains (Table 2). Phage isolates had neither activity against *S. aureus* DSM 105272 nor against *S. aureus* field strains from bovine udders.

**Table 2:** Phage titers (plaque-forming units, pfu/ml) and efficiency of plating (EOP).

		**PITT-1**	**PITT-2**	**PITT-3**	**PITT-4**	**PITT-5**	**PITT-6**	**PITT-7**	**PITT-8**	**PITT-9**	**PITT-10**	**PITT-11**
***S. hyicus***												
DSM 17421	Titer	– (a)	– (a)	– (a)	– (a)	– (a)	1.6 × 10^6^	2.8 × 10^6^	– (a)	– (a)	– (a)	– (a)
	EOP						2.1 × 10^−2^	2.8 × 10^−2^				
DSM 20459	Titer	– (a)	– (a)	– (a)	– (a)	– (a)	– (a)	2.3 × 10^6^	– (a)	– (a)	1.0 × 10^5^	1.0 × 10^5^
	EOP							2.3 × 10^−2^			4.0 × 10^−3^	2.4 × 10^−3^
07/2-4A	Titer	**1.3** **× ** **10**^**7**^	2.2 × 10^4^	8.3 × 10^5^	– (a)	– (a)	2.4 × 10^7^	9.4 × 10^7^	6.9 × 10^7^	2.9 × 10^7^	1.5 × 10^7^	5.1 × 10^7^
	EOP	**1**	1.7 × 10^0^	4.9 × 10^0^			3.2 × 10^−1^	9.4 × 10^−1^	3.8 × 10^−1^	4.8 × 10^−1^	6.0 × 10^−1^	1.2 × 10^0^
07/12-1A	Titer	– (a)	– (a)	– (a)	– (a)	– (a)	– (a)	– (a)	– (a)	– (a)	– (a)	– (a)
	EOP											
07/12-2A	Titer	8.2 × 10^6^	**1.3** **× ** **10**^**4**^	**1.7** **× ** **10**^**5**^	– (a)	– (a)	4.8 × 10^7^	9.6 × 10^7^	1.0 × 10^8^	2.9 × 10^7^	3.1 × 10^6^	6.2 × 10^6^
	EOP	6.3 × 10^−1^	**1**	**1**			6.3 × 10^−1^	9.6 × 10^−1^	5.6 × 10^−1^	4.8 × 10^−1^	1.2 × 10^−1^	1.5 × 10^−1^
83/7-1B	Titer	1.9 × 10^3^	– (a)	– (a)	**5.5** **× ** **10**^**6**^	**8.4** **× ** **10**^**6**^	1.5 × 10^4^	5.9 × 10^4^	1.2 × 10^3^	– (a)	2.8 × 10^2^	1.6 × 10^3^
	EOP	1.5 × 10^−4^			**1**	**1**	2.0 × 10^−4^	5.9 × 10^−4^	6.7 × 10^−6^		1.1 × 10^−5^	3.8 × 10^−5^
83/11-1A	Titer	6.8 × 10^6^	3.8 × 10^4^	7.2 × 10^5^	– (a)	– (a)	**7.6** **× ** **10**^**7**^	**1.0** **× ** **10**^**8**^	**1.8** **× ** **10**^**8**^	**6.1** **× ** **10**^**7**^	**2.5** **× ** **10**^**7**^	**4.2** **× ** **10**^**7**^
	EOP	5.2 × 10^−1^	2.9 × 10^0^	4.2 × 10^0^			**1**	**1**	**1**	**1**	**1**	**1**

*In bold: Host strain used for phage propagation*.

*– (a): Limit of detection: <10 pfu/ml*.

*No plaques were obtained on the following strains: S. epidermidis DSM 1798, S. epidermidis field strain L2– (a)82 (origin: plume), S. aureus DSM 105272, 5 S. aureus field strains (origin: bovine udder)*.

The case isolates of *S. hyicus* differed in their host properties: isolate 07/2-4A, 07/12-2A, and 83/11-1A were very efficiently lysed by most phages except PITT-4 and PITT-5. By contrast, 83/7-1B was not lysed by PITT-2, PITT-3, and PITT-9 and weakly lysed by all other phages except PITT-4 and PITT-5, which were isolated with 83/7-1B as the primary host. One of the isolates, 07/12-1A was resistant against all tested phages (Table 2). Host range was independent from the place of isolation: Phages isolated in one house showed good lytic activity against bacterial strains from the other house. The multispecies phage Twort (DSM 17442) had weak lytic activity for its *S. hyicus* host strain DSM 17421 (offered by the DSMZ for propagation), but no lytic activity in the DSM type strain or any of the field strains from this study (data not shown).

### Transmission Electron Microscopy

**Figure 1:**
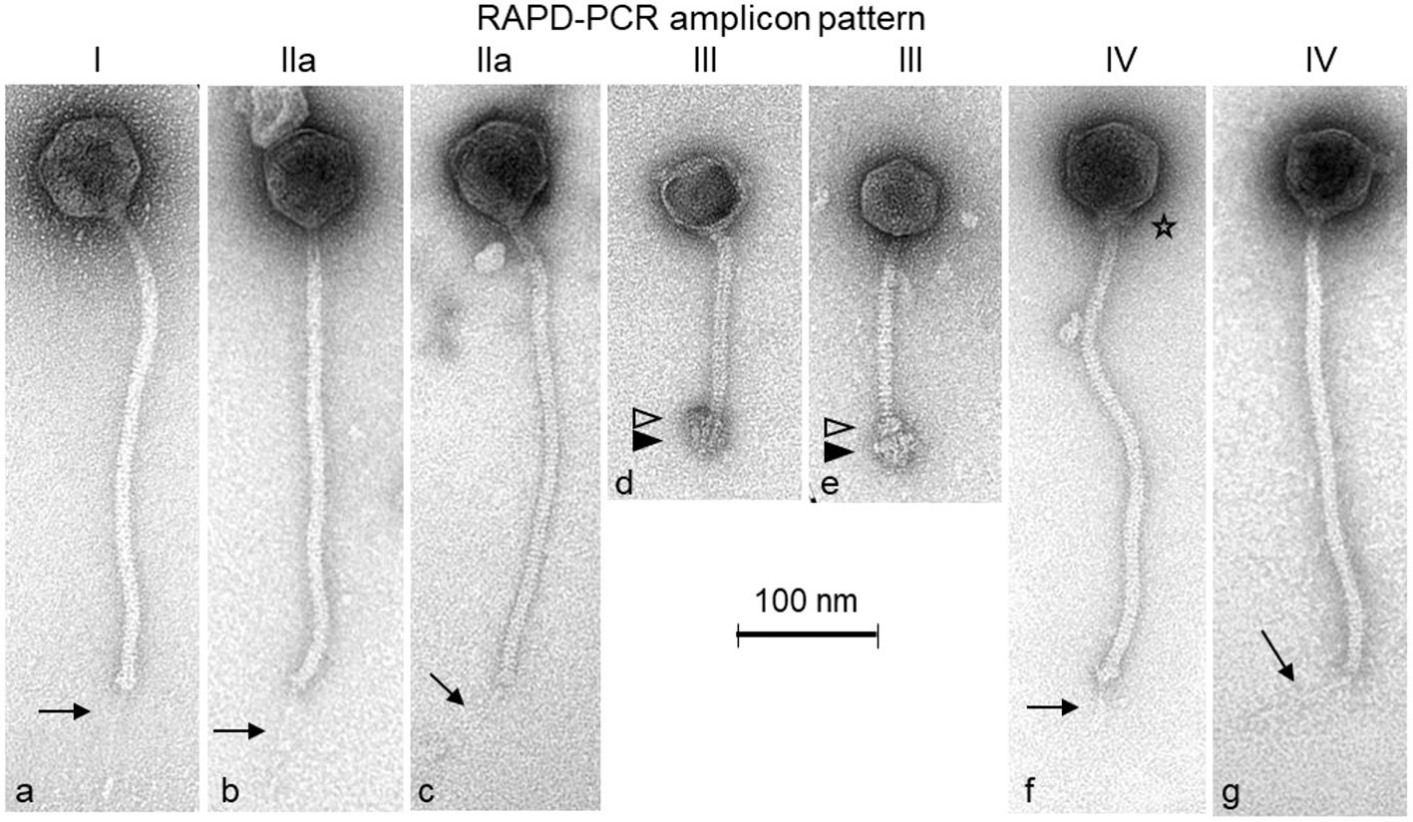
Transmission electron micrographs of the *S. hyicus* phages PITT-1 **(a)**, PITT-2 **(b)**, PITT-3 **(c)**, PITT-4 **(d)**, PITT-5 **(e)**, PITT-6 **(f)**, and PITT-7 **(g)** negatively stained with 2% (w/v) uranyl acetate. Structural details are indicated by arrows (faint distal central tail fibers), by open triangles (upper disc of complex baseplate structure), by filled triangles (lower disc of complex baseplate structure), and by an asterisk (collar/neck passage structure). All micrographs are shown with the same magnification (see 100-nm size reference bar). The RAPD-PCR patterns I, IIa, III-IV (see also Figure 2) are indicated on top of the micrographs.

By transmission electron microscopy, two different morphological groups of *Siphoviridae* phages could be distinguished (Figure 1). Nine phages (PITT-1-3 & 6-11) exhibit large isometric heads (diameter: 69–74 nm) and long, flexible tails (length: 323–346 nm). A notably thin tail fiber (length: 76–86 nm) is also visible under the distal end of the tail of phages PITT-1, 2, and PITT-7-11 (see arrows in Figures 1a–c,f,g). A unique morphological characteristic of phage PITT-6 is the presence of a distinct collar (i.e., neck passage structure) beneath the capsid (see asterisk in Figure 1f).

The second morphotype is represented by phages PITT-4 and PITT-5 (Figures 1d,e). These two *Siphoviridae* phages have smaller isometric heads (diameter: 59 nm) and shorter (147–160 nm) tails terminating with remarkably large and complex baseplate structures which are composed of a smaller upper baseplate disc (open triangles in Figures 1d,e) and a larger lower baseplate structure (filled triangles in Figures 1d,e). A bundle of short fiber-like appendages is attached below these baseplate discs. For phage PITT-4, phage particles with empty capsids could only be captured (Figure 1d).

### Genetic Diversity of Isolated Phages

#### RAPD-PCR

**Figure 2:**
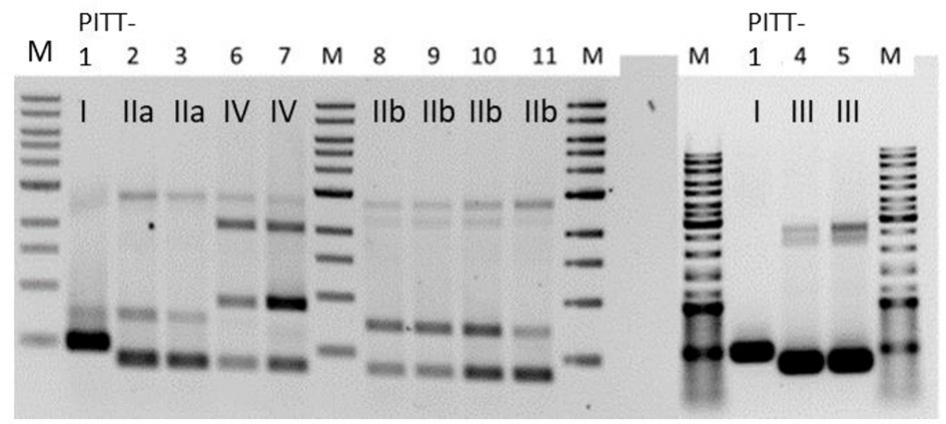
RAPD-patterns of *S. hyicus* phages PITT-1-11. Pattern I and II a/II b were exclusively found in samples from house 1. Pattern III was identified for 2 phages from both houses. The two phages with pattern IV were both detected in samples from house 2. PITT-1 is shown twice.

RAPD-PCR (Figure 2) revealed five different amplicon patterns: I (PITT-1), IIa (PITT-2,3), IIb (PITT-8,9,10,11), III (PITT-4,5), and IV (PITT-6,7). Pattern I was unique for a single phage isolate from house 1. Pattern IIa/b was the most common pattern representing phages from different primary bacterial hosts and samples (A, B). RAPD-patterns IIa (host 07/12-2A) and IIb (host 83/11-1A) differed in presence or absence of one band, while sharing three other bands. Notably, pattern II phages were only detected in house 1.

Due to differences in morphology and host range we expected the most pronounced differences between phages of pattern III and all other isolates, irrespective of their amplicon pattern. Thus, PITT-1 and PITT-5 were selected for sequencing.

#### Sequencing and Comparative Genomic Analysis

**Figure 3:**
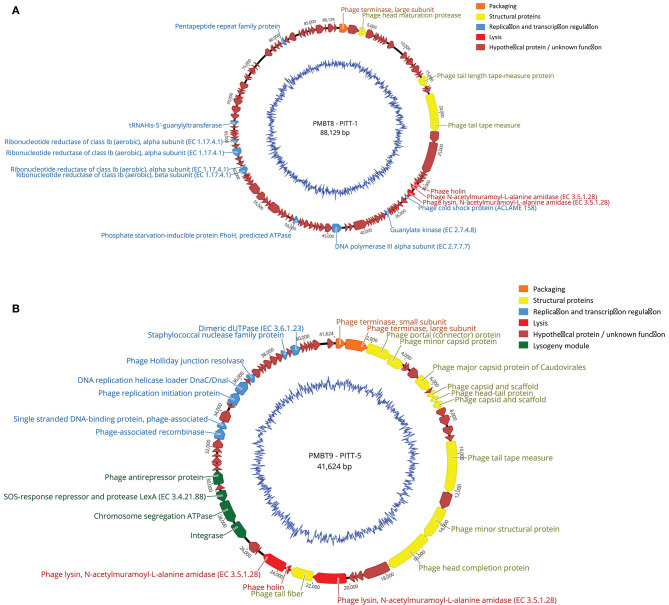
**(A,B)** Genome map of phages PITT-1 (PMBT8, 88,129 bp, **A**) and PITT-5 (PMBT-9, 41,624 bp, **B**) with structural and functional annotations. An automated annotation was obtained from PATRIC and all ORFs were subsequently manually annotated with BLASTP and SMART. Arrows indicate the predicted ORFs, which are labeled and color-coded based on their predicted function. The genomes are shown starting upstream of ORF1 that specifies the phage terminase large subunit. The ORFs involved in DNA packaging are marked in orange, the ORFs for structural proteins are marked in yellow and the ORF involved in lysis is marked in red. Blue arrows represent ORFs for phage DNA transcription and replication. Hypothetical ORFs and ORFs with no phage-related function are shown in dark red. The genome maps were generated using Geneious version 9.1.8.

The sequence of PITT-1 (PMBT8) resulted in a genome size of 88,129 bp with a mol% GC content of 31.6 and was deposited in the NCBI Genbank under acc. no. MK893987. PITT-5 (PMBT9) had a genome size of 41,624 bp and a mol% GC content of 36.2. Both phage genomes harbored no tRNA-encoding genes (Figures 3A,B). Using ResFinder, no acquired antibiotic resistance and virulence genes were identified in phage genomes.

The 88,129-bp genome of the virulent phage PITT-1 (PMBT8) comprises 122 putative open reading frames (ORFs) with 36 showing an assigned function and 86 hypothetical proteins (Figure 3A). An analysis of the PITT-1 genome with NCBI megablast resulted in only low similarities (coverage 8 and 6%; identity 78.99 and 78.92%) to the *S. epidermidis* phages vB_SepS_SEP9 (Melo et al., 2014) and 6ec (Aswani et al., 2014), respectively, which are members of the genus *Sextaecvirus* within the family *Siphoviridae*. Even when using less stringent searching conditions with discontiguous megablast, the coverage increased only to 29 and 32%, respectively, while identity decreased to 71%. With 32 and 71%, similar coverage and identity values were determined when comparing with the *S. aureus* phage VB-SauS-SA genome sequence. The multispecies *Staphylococcus* phage Twort, which can be propagated in *S. hyicus*, had only low similarity, with only 2% query coverage in the discontiguous megablast algorithm.

Protein blast (BLASTP) for assigned structural genes revealed a higher similarity between phage PITT-1 and *S. epidermidis* phages vB_SepS_SEP9 and 6ec, than comparisons based on overall nucleotide analysis. Similarities ranged from low similarities for the tape measure protein, i.e., 99% coverage and 42% identity for phage 6ec, 99% coverage and 41% identity for phage vB_SepS_SEP9, to higher similarities for the head maturation protein, i.e., 98% coverage and 74% identity (phage vB_SepS_SEP9) and 98% coverage and 67% identity (phage 6ec). However, using BLASTP, other proteins showed low similarities with a concurrent lower coverage, i.e., the holin protein which showed an identical coverage of 65% and identity of 45% for both *S. epidermidis* phages (vB_SepS_SEP9 and 6ec). Using BLASTP, no significant similarity was found between the assigned genes of PITT-1 and those of the multispecies *Staphylococcus* phage Twort.

The 41,624-bp genome of phage PITT-5 (PMBT9, deposited in GenBank under acc. MW221967; Figure 3B) revealed a moderate similarity to other phages in the databases: 37% coverage and 83.26% identity with the *S. aureus* phage EW, acc. AY954959 (Kwan et al., 2005), which belongs to the genus *Phietavirus* of the *Siphoviridae* phages.

Analysis of the protein sequences of the assigned structural proteins revealed high similarities to protein sequences deduced from the genomes of potential *Staphylococcus* prophages obtained from the databases. Moreover, some structural gene products also showed high similarities to proteins of isolated phages, like the head-tail protein (99% coverage, 90% identity to *Staphylococcus aureus* phage EW) or the capsid and scaffold protein (99% coverage, 95% identity to *Staphylococcus* phage SAP3). However, other structural proteins e.g., the tape-measure protein showed lower similarities (99% coverage, 64% identity to *Staphylococcus* phage IME1365_01, acc. no. KY653129). It is remarkable that this phage genome comprises an obviously intact lysogeny module with genes for an integrase, a SOS-response/LexA-like repressor and an antirepressor, indicating this phage still has the potential to integrate its genome into host strains.

#### Genetic Diversity of Host Bacteria

We also sequenced the genomes of three representative host bacteria, i.e., *S. hyicus* strains 07/2-4A, 83/7-1B, and 83/11-1A, respectively. These bacteria were the primary hosts used for enrichment of PITT-1, PITT-4 & 5, and PITT-6-11, respectively. SNP analysis revealed little diversity in the genomes of *S. hyicus* strains 07/2-4A and 83/11-1A suggesting they might be clonal strains, while *S. hyicus* strain 83/7-1B showed clear differences to those strains (Table 3). Additionally, sequencing resulted in the identification of two different *exh* virulence genes for these isolates, namely *exh*A in 07/2-4A and 83/11-1A, and *exh*C in 83/7-1B.

**Table 3:** SNP matrix of *S. hyicus strains*.

SNP^*^ distance	**07/2-4A**	**83/11-1A**	**83/7-1B**
07/2-4A	0	15	5,226
83/11-1A	15	0	5,227
83/7-1B	5,226	5,227	0

* *Single nucleotide polymorphism*.

## Discussion

Since EE is not under systematic control regulated by European law or by the OIE, there is a lack of monitoring data and the prevalence is unknown. However, the Iowa State University states that EE occurs “in most major swine raising countries (…), one case occasionally seen by most swine practitioners.” (https://vetmed.iastate.edu/vdpam/FSVD/swine/index-diseases/greasy-pig). Farm outbreaks can affect a vast number of animals: in a German case of farm shutdown required by local authorities, 258 pig pens were simultaneously affected by EE according to media reports based on interviews with official authorities [(https://www.swr.de/-/id=14731830/property=download/nid=233454/16d4gu4/droht-ferkelzuechter-straathof-das-aus.pdf), last access 2020.11.27]. It is important to note that the causative agent of EE, coagulase-variable *S. hyicus*, episodically also causes zoonotic disease in humans (Casanova et al., 2011). There is no approved vaccination against greasy pig disease; farm-specific vaccines are applied to control local outbreaks [StIKo-Vet, FLI Germany, (https://stiko-vet.fli.de/de/aktuelles/)] and antibiotics are used to treat acute disease. Often, penicillin G is the drug of choice, although susceptibility results contradict this empiric tradition (Park et al., 2013a). Bacteriophages might offer interesting alternatives for individual topical treatment, as well as for sanitation of the environment. Despite current restrictive approval of bacteriophages in the EU, Fernández et al. (2018) are convinced that it is “only a matter of time that (…) drawbacks can be overcome.” Thus, the detection and proper characterization of new bacteriophages is of common interest. We isolated a set of 11 *S. hyicus* phages (belonging to 2 morphologically different groups of *Siphoviridae*) from the same piglet farm suffering from EE. Host *S. hyicus* bacteria and bacteriophages were spread between two different pig houses. Bacteriophages were most frequently detected when samples were taken by rinsing diseased pigs with tap water.

In our study, at least two different *S. hyicus* strains were isolated from clinically affected animals on the same farm. Both strains carried different virulence genes of the *exh*-type (*exh*A / *exh*C). In a Danish study on 314 isolates from 60 cases of EE, *exh*A (20%) and *exh*C (18%) were also prevalent among virulent strains, although *exh*B (33%) was the most common virulence gene (Andresen and Ahrens, 2004). However, distribution of different *exh*-genes differs by geography (González-Martín et al., 2020), with *exh*A being the most prevalent *exh*-gene in Japan in 2007 (Futagawa-Saito et al., 2007). In our study, *S. hyicus* strains with different *exh*-genes differed in their susceptibility to phages: while the *exh*A-positive strains 07/2-4A and 83/11-1A were efficiently lysed by all but two phages, the *exh*C-positive strain 83/7-1B was only weakly lysed by most phages, except PITT-4 and PITT-5. The occurrence of at least two different, but virulent *S. hyicus* strains on the same farm thus would have implications for herd-specific phage application, but also for farm-specific vaccination.

Sequencing of two phages as representatives of the two different morphotypes revealed that the lytic phage PITT-1 (PMBT8, acc. No. MK893987) had very little resemblance with multispecies *Staphylococcus* phage Twort, the only *S. hyicus*-effective phage with a sequence entry in the NCBI Genbank). The sequence identity was higher to *S. epidermidis Siphoviridae* phages (vB_SepS_SEP9 and 6ec; Aswani et al., 2014; Melo et al., 2014), but with a query coverage still as low as 29–32%. Despite their genetic diversity, these three phages showed a very similar morphology—icosahedral heads and elongated flexible tails. This may be associated with the higher similarity observed for blastp analysis of specific structural proteins when compared to the overall phage nucleic acid sequence analysis. PITT-1 revealed the largest head [74 nm compared to 64 nm (SEP9) and 69 nm (6ec)] but the shortest tail [346 nm compared to 375 nm (SEP9) and 362 nm (6ec)]. A second phage, PITT-5, was sequenced, which differed from phage PITT-1 with respect to morphology, narrow host range and DNA-sequence. The phage PITT-5 may not be well-adapted to lytic propagation, as it reveals only low (or no) lytic activity against all of the investigated host strains, except for its primary host.

Notably, only few bacteriophages specific for strains of coagulase-negative or -variable *Staphylococcus* species have been characterized in detail, and the number of recorded phages for coagulase-negative staphylococci was reported to be below 10 in 2014 (Melo et al., 2014). Historically, phages for *S. hyicus* had been repeatedly described for the purpose of phage typing (Hájek and Horák, 1981; Kawano et al., 1983; Kibenge et al., 1983; Shimizu et al., 1987a,b; Wegener, 1993a,b). However, no further characteristics (e.g., morphologies or sequence data) of these phages are publicly available. In contrast to our host range assessment, the host range of some of these phages comprised also strains of *S. epidermidis* (Kawano et al., 1983), as well as strains of *S. intermedius* (which were not tested in our study).

The phage PITT-5 appears to be a temperate phage and is therefore probably not suitable for therapeutic or sanitation applications. Thus, the strictly lytic nature of PITT-1 makes this phage a promising candidate for further studies, which should include *in vivo* trials. The lack of antibiotic resistance genes and virulence genes encourages further investigations on phage PITT-1. However, the diversity of virulent *S. hyicus* strains isolated in the same farm environment, might require the application of blends of phages with different host ranges.

## Data Availability Statement

The datasets presented in this study can be found in online repositories. The names of the repository/repositories and accession number(s) can be found in the article.

## Ethics Statement

Ethical review and approval was not required for the animal study because samples were taken for the purpose of routine veterinary diagnostics, which does not need any ethical approval according to national and European law. Written informed consent was obtained from the owners for the participation of their animals in this study.

## Author Contributions

JT conceived and described the isolation of bacteriophages and bacteria. JT and SS conceived and described the cultural experiments. SS described the molecular characterization of the bacteriophages. GT performed and described the isolation of bacteriophages, bacteria, and the cultural experiments. EB performed the genomic characterization. EB and SS created and described the sequencing data and the genome maps. NW and HN conceived, performed, and described the electron microscopy analysis. CH conceived the isolation of bacteriophages, bacteria, the cultural experiments, and wrote the discussion. CF conceived the genomic characterization. CH and CF conceived the structure of the manuscript. All authors commented on the first drafts of the manuscript and contributed to the final version.

## Conflict of Interest

The authors declare that the research was conducted in the absence of any commercial or financial relationships that could be construed as a potential conflict of interest.
